# Significance of Hydroxyl Groups on the Optical Properties of ZnO Nanoparticles Combined with CNT and PEDOT:PSS

**DOI:** 10.3390/nano12193546

**Published:** 2022-10-10

**Authors:** Keshav Nagpal, Erwan Rauwel, Elias Estephan, Maria Rosario Soares, Protima Rauwel

**Affiliations:** 1 Institute of Forestry and Engineering, Estonian University of Life Sciences, 51014 Tartu, Estonia; 2 LBN, University of Montpellier, 34193 Montpellier, France; 3 CICECO, University of Aveiro, 3810-193 Aveiro, Portugal

**Keywords:** ZnO, ZnO-CNT, ZnO-PEDOT:PSS, nanoparticles, hybrids, hydroxyl groups, non-aqueous sol–gel, surface defects, photoluminescence

## Abstract

We report on the synthesis of ZnO nanoparticles and their hybrids consisting of carbon nanotubes (CNT) and polystyrene sulfonate (PEDOT:PSS). A non-aqueous sol–gel route along with hydrated and anhydrous acetate precursors were selected for their syntheses. Transmission electron microscopy (TEM) studies revealed their spherical shape with an average size of 5 nm. TEM also confirmed the successful synthesis of ZnO-CNT and ZnO-PEDOT:PSS hybrid nanocomposites. In fact, the choice of precursors has a direct influence on the chemical and optical properties of the ZnO-based nanomaterials. The ZnO nanoparticles prepared with anhydrous acetate precursor contained a high amount of oxygen vacancies, which tend to degrade the polymer macromolecule, as confirmed from X-ray photoelectron spectroscopy and Raman spectroscopy. Furthermore, a relative increase in hydroxyl functional groups in the ZnO-CNT samples was observed. These functional groups were instrumental in the successful decoration of CNT and in producing the defect-related photoluminescence emission in ZnO-CNT.

## 1. Introduction

Hybrid nanocomposites combining organic and inorganic counterparts have a multitude of applications, e.g., light-emitting diodes (LED), solar cells, and photodetectors [1,2,3]. Organic materials consist of polymers possessing remarkable properties, such as easy processing, flexibility, and good conductivity [4]. However, their high cost and lack of stability are obstacles for practical devices. On the other hand, inorganic materials present higher structural, chemical, and functional stability, as well as a high charge mobility, making them suitable for optoelectronic applications [5]. Therefore, the combination of organic with inorganic materials provides robust multifunctional nanocomposites with applications in flexible electronic and photonic devices [6,7,8].

Conducting polymers such as poly (3,4-ethylenedioxythiophene) poly(styrenesulfonate) (PEDOT:PSS) are already being incorporated into organic thin film transistors, organic LED, organic solar cells, capacitors, batteries, and thermoelectric devices, as well as technologies such as touch screens and electronic papers [9,10]. In addition, PEDOT:PSS is mechanically stable and highly flexible. Various combinations of PEDOT:PSS with inorganic materials, such as SnO_2_, TiO_2_, CdS, CdSe, ZnO and metal nanostructures, have been investigated to that end [11,12]. Among these inorganic nanomaterials, ZnO is promising due its wide band gap of 3.37 eV, large exciton binding energy of 60 meV, high chemical stability, and remarkable electrical and optical properties [13]. Moreover, the high surface-to-volume ratio of ZnO nanoparticles implies a spontaneous presence of surface defects, including oxygen vacancies (V_O_), oxygen interstitials (O_i_), and zinc interstitials (Zn_i_). Therefore, in addition to the UV emission, known as the near-band emission (NBE), ZnO nanoparticles emit within the entire visible spectrum, also known as defect-level emission (DLE) [14]. The latter depends on both the surface and the volume defects introduced during the synthesis of the nanoparticles [15,16]. For example, ZnO nanoparticles prepared by aqueous sol–gel routes tend to emit higher NBE and a negligible DLE [17]. On the other hand, for ZnO nanoparticles prepared by non-aqueous sol–gel routes, the emission depends on the presence of hydrates in the precursor [18,19]. In fact, hydrates in the precursor contribute to the enhancement of NBE due to improved oxidation of ZnO during synthesis [18]. On the other hand, adsorption of hydroxyl groups on the surface of ZnO nanoparticles has been shown to increase the visible PL emission [20]. The chemisorption of oxygen radicals from air on the surface of ZnO nanoparticles also augments green emission from them [21]. In general, small nanoparticles possess a high surface-to-volume ratio, and therefore harbor higher amounts of surface defects. For larger ZnO nanoparticles, defects can be both surface and volume related [22]. 

Recently, ZnO-CNT nanohybrids have attracted considerable interest due to their high stability and superior photonic, electrochemical and electromagnetic properties, which originate from interfacial effects. In a previous study, we successfully passivated ZnO surface states by combining them with CNT via sonication [17]. In this work, we carry out ZnO nanoparticle synthesis via a non-aqueous sol–gel route with hydrated (zinc acetate dihydrate (Zn(CH_3_CO_2_)_2_.2H_2_O) and anhydrous (zinc acetate anhydrous (Zn(CH_3_CO_2_)_2_) precursors. In a study by Šarić et al., it was shown that with similar precursors, ZnO precipitation could be promoted through an esterification reaction that generates water upon the addition of acetic acid [23]. In our reaction, only absolute ethanol is used as a solvent. It plays a crucial role in controlling the size and shape of ZnO nanoparticles, and consequently, in the formation of various surface defects. Due to the addition of sodium hydroxide in this work, the basic character of the solution prevents any esterification reaction and in turn, no water molecules are formed. This route therefore enables the formation of very small ZnO nanoparticles functionalized with hydroxyl groups, promoting the decoration of CNT with ZnO. We then decorated CNT with ZnO nanoparticles in order to create a hybrid nanocomposite. Subsequently, we fabricated a second type of hybrid nanocomposite consisting of ZnO-PEDOT:PSS and compared the evolution of the surface defects to ZnO-CNT. These optical properties are discussed in terms of synthesis conditions, crystal structure, chemical properties, and the morphology of ZnO nanoparticles and their hybrids. The objective is to use these hybrid materials in LED. Therefore, finding a way to control these surface defects or trap states for LED applications is a priority, as they are detrimental to device properties.

## 2. Materials and Methods

### 2.1. Synthesis

#### 2.1.1. ZnO

Two different zinc precursors, Zn(CH_3_CO_2_)_2_.2H_2_O (99.5%, Fisher Scientific, Loughborough, UK) and Zn(CH_3_CO_2_)_2_ (99.9%, Alfa Aesar, Kandel, Germany), were used for the synthesis of ZnO nanoparticles via non-aqueous sol–gel routes. Sodium hydroxide (NaOH) (99.9%, Aldrich) was used as a reducing agent. All the chemicals used were of analytic reagent grade. To prepare 0.05 M solutions of zinc precursors, 219.5 mg of Zn(CH_3_CO_2_)_2_.2H_2_O or 183.48 mg of Zn(CH_3_CO_2_)_2_ were dissolved in 20 mL absolute ethanol in a beaker placed in a water bath. The solutions were maintained at 65 °C under continuous magnetic stirring until the precursors were completely dissolved in absolute ethanol. Furthermore, a solution of 0.10 M NaOH in 20 mL absolute ethanol was prepared. The NaOH solution was added dropwise to the zinc precursor solutions. Thereafter, the mixtures were maintained at 65 °C for 2 h after which they were cooled to ambient temperature. White ZnO precipitates settled at the bottom of the reaction vessel. The resulting solutions containing ZnO nanoparticles were then centrifuged at 4500 rpm for 6 min, followed by drying for 24 h in air at 60 °C. This resulted in an agglomeration of ZnO nanoparticles in the form of a pellet, which is typical after drying nanoparticles synthesized via sol–gel routes. These pellets were thereafter crushed using a pestle and mortar to obtain a very fine powder of ZnO nanoparticles. 

#### 2.1.2. ZnO-CNT Hybrids

For the preparation of ZnO-CNT hybrids, firstly, a solution of CNT was prepared by mixing 4 mg of CNT in 50 mL absolute ethanol and sonicating until a homogenous mixture was obtained. As before, 0.05 M zinc precursor solutions were prepared in 20 mL absolute ethanol. To prepare 0.10 M NaOH solutions, ~80 mg of NaOH was added to 19 mL absolute ethanol, in which 1 mL CNT mixture (~0.08 mg) was added. The final mixtures were sonicated and added dropwise to zinc precursor solutions. Thereafter, the reaction was completed as described earlier, and ZnO-CNT hybrid pellets were obtained. These pellets were gently crushed to obtain fine black powders of ZnO-CNT nanohybrids. 

#### 2.1.3. ZnO-PEDOT:PSS Hybrids

For the preparation of ZnO-PEDOT:PSS hybrids, 20 mg of the as-synthesized ZnO nanoparticles were taken, to which 400 mg of PEDOT:PSS (as purchased) was added. The mixtures were sonicated for 1 h and dried at 70 °C for 24 h. The dried mixtures were further gently crushed to obtain blue powders with agglomerated particles of ZnO-PEDOT:PSS nanohybrids. 

### 2.2. Characterization

X-ray diffraction patterns were collected in Bragg–Brentano geometry using a Bruker D8 Discover diffractometer (Bruker AXS, Germany) with CuKα1 radiation (λ = 0.15406 nm) selected by a Ge (111) monochromator and LynxEye detector. Transmission electron microscopy (TEM) was carried out on a Tecnai G2 F20 (Netherlands) is a 200 kV field emission gun (FEG) for high-resolution and analytical TEM/STEM. It provided a point-to-point resolution of 2.4 Å. XPS measurements were performed at room temperature with a SPECS PHOIBOS 150 hemispherical analyzer (SPECS GmbH, Berlin, Germany)) with a base pressure of 5 × 10^−10^ mbar using monochromatic Al K alpha radiation (1486.74 eV) as excitation source operated at 300 W. The energy resolution as measured by the FWHM of the Ag 3d_5/2_ peak for a sputtered silver foil was 0.62 eV. The spectra were calibrated with respect to the C1s at 284.8 eV. The optical absorbance of ZnO, ZnO-CNT and ZnO-PEDOT:PSS nanohybrids was determined using a NANOCOLOR UV-VIS II spectrometer (MACHEREY-NAGEL, Germany) in the 200–900 nm region. The band gap of ZnO, ZnO-CNT and ZnO-PEDOT:PSS nanohybrids was subsequently calculated using Tauc plots. PL spectroscopy was carried out at room temperature with an excitation wavelength of 365 nm of an LSM-365A LED (Ocean Insight, USA) with a specified output power of 10 mW. The emission was collected by FLAME UV-Vis spectrometer (Ocean optics, USA) with a spectral resolution 1.34 nm. Optical images of ZnO were taken under a UV lamp ZLUV220 (China) with an excitation source of 365 nm. Raman spectra were collected using a WITec Confocal Raman Microscope System alpha 300R (WITec Inc., Ulm, Germany). Excitation in confocal Raman microscopy is generated by a frequency-doubled Nd:YAG laser (New-port, Irvine, CA, USA) at a wavelength of 532 nm, with 50 mW maximum laser output power in a single longitudinal mode. The system was equipped with a Nikon (Otawara, Japan) objective with a X20 magnification and a numerical aperture NA = 0.46. The acquisition time of a single spectrum was set to 0.5 s. 

## 3. Results

Table 1 provides a list of ZnO, ZnO-CNT and ZnO-PEDOT:PSS samples synthesized in this work. Samples ZnO-D and ZnO-A correspond to ZnO nanoparticles synthesized from Zn(CH_3_CO_2_)_2_.2H_2_O and Zn(CH_3_CO_2_)_2_ precursors, respectively. Samples ZnO-D-CNT, ZnO-A-CNT, ZnO-D-PEDOT:PSS and ZnO-A-PEDOT:PSS correspond to the hybrids of samples ZnO-D and ZnO-A with CNT and PEDOT:PSS, respectively. In this study, the terms ZnO samples refer to samples ZnO-D and ZnO-A; ZnO-CNT hybrids refer to samples ZnO-D-CNT and ZnO-A-CNT and ZnO-PEDOT:PSS hybrids refer to samples ZnO-D-PEDOT:PSS and ZnO-A-PEDOT:PSS. 

### 3.1. Structure and Morphology

**Figure 1:**
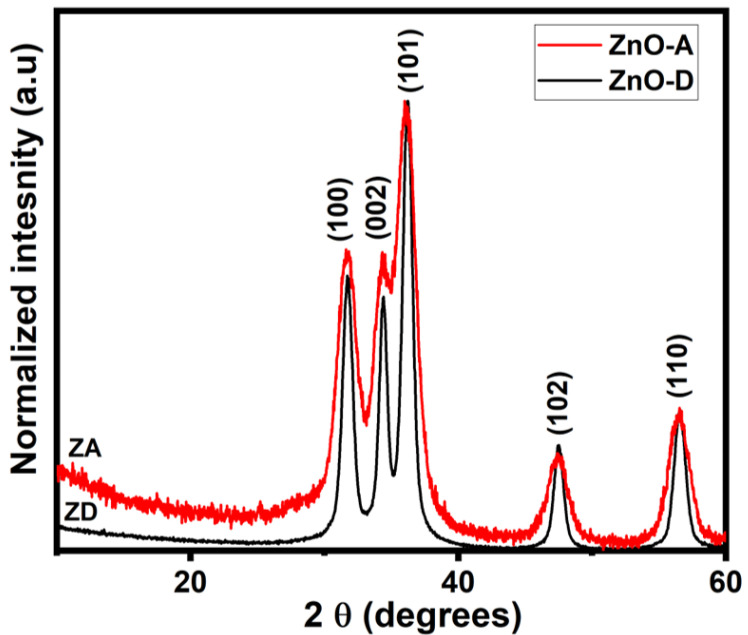
Normalized XRD patterns of samples ZnO-D and ZnO-A.

The XRD patterns of ZnO samples ZnO-D and ZnO-A are shown in Figure 1. The peaks (100), (002), (101), (102), and (110) correspond to the hexagonal Wurtzite structure (a = 3.25 Å and c = 5.20 Å) of ZnO (JCPDS, Card Number 36-1451). No secondary phases are visible in the XRD patterns, indicating that single-phase ZnO nanoparticles were formed. In addition, XRD patterns illustrate that both samples ZnO-D and ZnO-A exhibit very small particle sizes due to broader XRD peaks. The size of nanoparticles was estimated using the Scherrer equation [24].

(1) $$D=\frac{0.9\lambda }{\beta \;cos\theta }$$

where D is particle size, λ (=0.15406 nm) is the wavelength of incident X-ray beam, β is FWHM in radians, and θ is Bragg’s diffraction angle. Size calculation was carried out by considering the highest-intensity (101) peak. The calculated ZnO nanoparticle sizes of samples ZnO-D and ZnO-A were ~9 nm and ~5 nm, respectively. However, the actual size and shape of ZnO nanoparticles were confirmed from TEM studies as discussed below.

**Figure 2:**
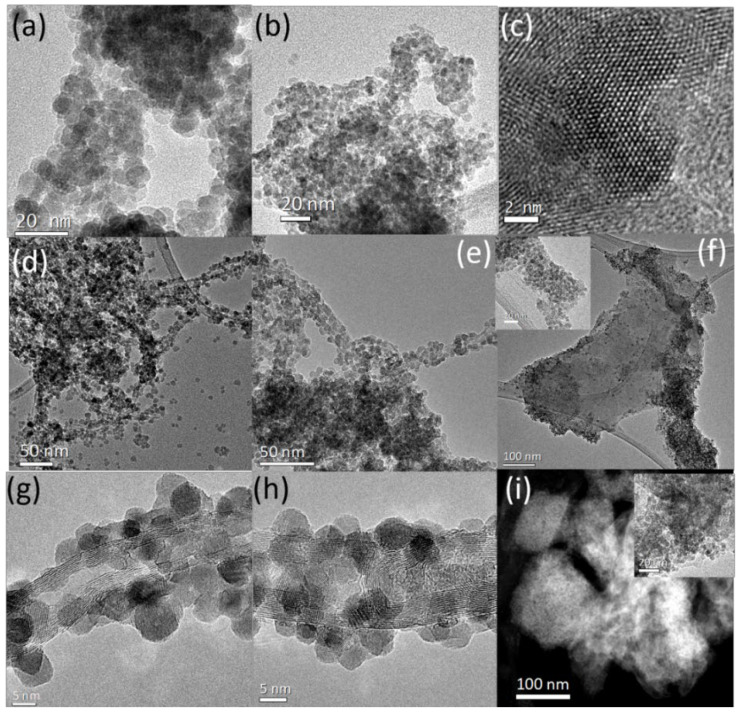
Overview TEM images of samples (**a**) ZnO-D, (**b**) ZnO-A, (**c**) HRTEM image of ZnO-A nanoparticles. Overview TEM images of (**d**) ZnO-D-CNT, (**e**) ZnO-A-CNT, (**f**) ZnO-D-PEDOT:PSS. HRTEM images of (**g**) ZnO-D-CNT, (**h**) ZnO-A-CNT and (**i**) STEM image of ZnO-A-PEDOT:PSS.

The morphological features of the as-synthesized ZnO samples, ZnO-CNT hybrids and ZnO-PEDOT:PSS hybrids were studied by TEM, as shown in Figure 2. TEM images in Figure 2a,b consist of overviews of the as-grown samples ZnO-D and ZnO-A, respectively. The micrographs reveal spherical nanoparticles of uniform size that tend to agglomerate. With the help of size distribution histograms of the as-synthesized ZnO samples, we estimate an average nanoparticle size of ~5.2 nm and ~4.8 nm for ZnO-D and ZnO-A, respectively. Figure 2c is a high-resolution TEM (HRTEM) image of sample ZnO-A, where two ZnO nanoparticles are oriented along the [0001] zone axis of the basal plane of the Wurtzite structure. Figure 2d,e are low-magnification TEM images of the samples ZnO-D-CNT and ZnO-A-CNT, respectively. ZnO nanoparticles dominate the TEM images due to the low wt% (~1 wt%) of CNT in the samples. HRTEM images of ZnO-CNT are presented in Figure 2g,h. The walls of the CNT are clearly visible along with nanoparticles decorating them. We observe that the presence of CNT does not alter the crystallinity or the size distribution of the nanoparticles, and average sizes of ~5.7 nm and ~4.7 nm were retained. The micrographs therefore clearly indicate successful decoration of the nanoparticles on the walls of the CNT. In our study, the nanotubes were functionalized by sonication in pure ethanol; hence, the most likely functional groups present are carboxyl (COOH) that can be broken down into carbonyl (C-O) and hydroxyl (OH) [25]. These functional groups promote a covalent bonding between the CNT and ZnO nanoparticles, necessary for the decoration of CNT. Figure 2f,i are the TEM and scanning transmission electron microscopy (STEM) images of ZnO-D-PEDOT:PSS and ZnO-A-PEDOT:PSS samples, respectively. PEDOT:PSS appears as flakes without any noticeable agglomeration of ZnO nanoparticles in the polymer layer. However, some areas of PEDOT:PSS are more densely packed with ZnO nanoparticles. The insets of Figure 2f,i are high-magnification TEM images of the samples emphasizing on their homogeneous distribution in the PEDOT:PSS matrix.

**Figure 3:**
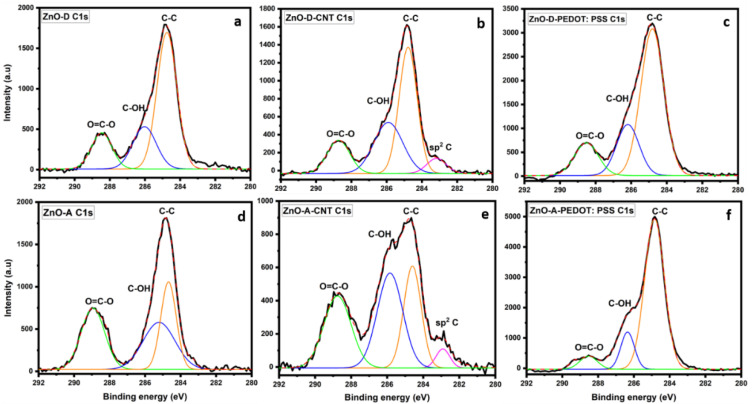
High-resolution XPS spectra of the C1s region of (**a**) ZnO-D, (**b**) ZnO-D-CNT, (**c**) ZnO-D-PEDOT:PSS, (**d**) ZnO-A, (**e**) ZnO-A-CNT and (**f**) ZnO-A-PEDOT:PSS.

**Figure 4:**
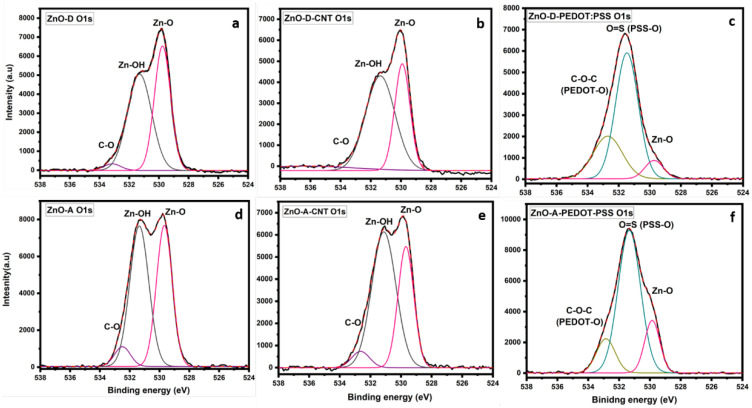
High-resolution XPS spectra of the O 1s region, (**a**) ZnO-D, (**b**) ZnO-D-CNT, (**c**) ZnO-D-PEDOT:PSS, (**d**) ZnO-A, (**e**) ZnO-A-CNT and (**f**) ZnO-A-PEDOT:PSS.

The high-resolution XPS spectra of the C 1s and O 1s regions of the as-synthesized and hybrid ZnO nanoparticles are shown in Figure 3 and Figure 4, respectively. For the C 1s spectra of the as-synthesized samples in Figure 3a,d, the photoelectron peak at 284.8 eV corresponds to adventitious carbon [26]. Several carbon bonds are present in the samples, such as C-OH, O=C-O originating from the NaOH and acetate precursors used in the syntheses [27]. Both ZnO-A and ZnO-D contain oxygen and hydroxyl groups that are chemisorbed. The C 1s region of ZnO-CNT in Figure 3b,e manifests an additional peak corresponding to sp^2^ hybridization of C atoms in the CNT at a binding energy of 283 eV. In addition, the C-OH peak is relatively more intense for the ZnO-A-CNT sample compared to the as-synthesized ZnO sample in Figure 3d, which could indicate an increase of hydroxyl groups or oxygen vacancies [18]. In fact, sonication of CNT in ethanol engenders a breakdown of the sidewalls, which then produces C-dangling bonds [28]. After sonication and during the initial stages of synthesis, CNT were mixed in ethanol and heated to a temperature of 65 °C for 2 h during which a solution of NaOH was added dropwise. Considering the hydroxyl rich conditions, the attachment of OH groups to C-dangling bonds is likely. For the ZnO-D-based samples, in Figure 3a–c, the relative intensities of the various peaks in the C1s region are similar, unlike the ZnO-A-based samples. In addition, in Figure 3f, a decrease in the O=C-O and C-OH peak intensities relative to the C-C peak for sample ZnO-A-PEDOT:PSS is observed, indicating an oxygen-deficient or -reduced PEDOT:PSS polymer.

**Figure 5:**
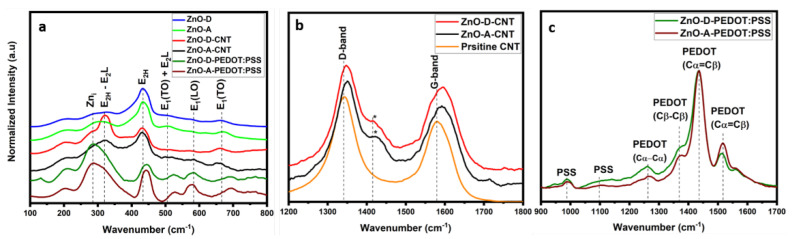
Normalized Raman spectra of (**a**) ZnO-D, ZnO-A, ZnO-D-CNT, ZnO-A-CNT, ZnO-D-PEDOT:PSS and ZnO-A-PEDOT:PSS from 100-800 cm^−1^, (**b**) pristine CNT, ZnO-D-CNT and ZnO-A-CNT from 1200-1800 cm^−1^, and (**c**) ZnO-D-PEDOT:PSS and ZnO-A-PEDOT:PSS from 900–1600 cm^−1^.

The high-resolution spectra of the O 1s region of the ZnO samples, ZnO-CNT hybrids and ZnO-PEDOT:PSS hybrids in Figure 4 consist of several peaks, including lattice oxygen peak of ZnO or the Zn-O bond. Additionally, for the samples ZnO-D (Figure 3a), ZnO-D-CNT (Figure 3b), ZnO-A (Figure 3d) and ZnO-A-CNT (Figure 3e), photoelectron peaks that correspond to hydroxyl groups are also visible. In particular, the photoelectron peak at around 531.5 eV is attributed to Zn-OH bonds as well as oxygen vacancies [22]. TEM analysis estimated an average ZnO nanoparticle size of 5 nm, implying a very high surface-to-volume ratio. In such small nanoparticles, surface oxygen vacancies are prevalent. Since the C 1s region contains oxygen or hydroxyl components and the O 1s region contains carbon and hydroxyl components, it therefore suggests that hydroxyl groups are responsible for the decoration of CNT with ZnO. This directly implies that hydroxyl groups enable the anchoring of ZnO on CNT surface through covalent bonding with carbon, as there is no indication of Zn-C bonds. TEM images clearly indicate that ZnO nanoparticles grow directly on the CNT sidewalls through Zn-O/OH-C bonds. Furthermore, the O 1s region of the ZnO-PEDOT:PSS hybrids of Figure 4c,f display additional peaks, along with differences in relative intensities of peaks compared to ZnO and ZnO-CNT hybrids. In these samples, the characteristics of the PEDOT:PSS polymer is more dominant. In fact, two peaks—C-O-C of PEDOT at 532.7 eV and O=S of PSS at 531.7 eV—are visible as well as a third peak of Zn-O [29]. In general, the ZnO-D lattice, i.e., as-synthesized ZnO-D or ZnO-D in the nanohybrids, shows a more stable oxygen component, when considering the C 1s spectra of Figure 5a–c, where the relative intensities of O=C-O, C-OH and C-C peaks are rather constant. However, for the ZnO-A-PEDOT:PSS, the PEDOT peak is less intense than the PSS peak. In fact, PEDOT:PSS macromolecule consists of PEDOT that is positively charged, highly conductive, and hydrophobic. On the other hand, PSS is negatively charged, insulating and hydrophilic. If we consider that the nanoparticles were dispersed in an aqueous solution of PEDOT:PSS, then the adsorption of hydroxyl groups on the surface of the ZnO nanoparticles is inevitable. From the relative intensities of various peaks of the O1s region in Figure 4a,d, ZnO-A tends to adsorb a higher quantity of hydroxyl groups than ZnO-D. Consequently, the surface of ZnO-A is more electronegative with a propensity to the positively charged PEDOT. The O 1s region of ZnO-A-PEDOT:PSS consists of a less intense C-O-C peak and a highly intense O=S peak compared to ZnO-D-PEDOT:PSS. The reduction in the relative intensity of the C-O-C peak suggests that either PEDOT was removed or degraded on adding ZnO-A. In addition, the shift in the Zn-O and C-O-C peaks to higher binding energies confirms the formation of a covalent bond between the C of PEDOT and OH groups present on the ZnO surface. The higher binding energy of the Zn-O peak along with an increase in its intensity indicates that the configuration for the lattice oxygen of ZnO-A becomes more stable.

The vibrational properties of the ZnO nanoparticles and their hybrids were investigated using Raman spectroscopy. The results obtained from Raman spectroscopy complement those obtained via XPS. In fact, chemical and structural changes can be evaluated simultaneously on ZnO and CNT or PEDOT:PSS using Raman spectroscopy. Figure 5a compares the different vibrational modes obtained from these samples in the range of 100–800 cm^−1^. The first-order phonon modes obtained at ~440 cm^−1^, ~585 cm^−1^ and ~667 cm^−1^, correspond to E_2H_, E_1_ (LO) and E_2_ (TO) modes, respectively [21]. Other modes obtained at ~320 cm^−1^ and 506 cm^−1^ are multiphonon scattering modes that correspond to the E_2H_–E_2L_ and E_1_(TO) + E_2L_ modes, respectively [30]. The E_2H_, E_2H_–E_2L_, E_1_ (LO) modes involve the oxygen component of ZnO. More specifically, the E_2H_ at 440 cm^−1^ corresponds to lattice oxygen, whereas the E_1_ (LO) corresponds to oxygen-related defects [31]. For all the samples, the E_2H_ mode intensities are high, implying that the ZnO lattice structure is unaffected on hybridizing with CNT or PEDOT:PSS. However, the relative intensity of the E_1_ (LO) band increases in the nanocomposites, indicating an increased number of surface defects [32]. The attachment of ZnO on the sidewalls of the CNT through hydroxyl functional groups indicates that the interfacial region and, therefore, the surface of ZnO are highly defective. Additionally, the E_2H_ peak for ZnO-PEDOT:PSS samples has shifted to a higher wavenumber of 445 cm^−1^ owing to chemical interactions between PEDOT and ZnO. This peak is more intense for ZnO-A-PEDOT:PSS than ZnO-D-PEDOT:PSS, which once again supports that ZnO-A has a more stable lattice configuration in PEDOT:PSS. 

Raman signatures lower than 300 cm^−1^ are assigned to the vibrations of Zn_i_, and those above 300 cm^−1^ are assigned to the vibrations of oxygen atoms [33]. The peak at 275 cm^−1^ has been attributed to Zn_i_ or Zn_i_ clustering [34,35]. The intensity of this mode increases relative to the other modes in the CNT-based nanocomposites and is the highest for PEDOT:PSS-based nanocomposites. This suggests that the amount of Zn_i_ is higher than Vo in the hybrid samples. Another mode at 526 cm^−1^ is observed for the PEDOT:PSS nanocomposites, corresponding to the combination of Vo and Zn_i_ [34]. A lower-intensity peak at the same localization is also visible in the ZnO-CNT-based samples. In general, the relative intensity of this combined mode increases in the hybrid samples owing to an increase in Zni. In Figure 5b, Raman bands from 1200–1800 cm^−1^ of pristine CNT are compared to those of ZnO-CNT. The D-band at 1341 cm^−1^ for pristine CNT redshifts for ZnO-CNT to ~1351 cm^−1^. A similar redshift in the G-band from 1579 cm^−1^ to ~1592 cm^−1^ is also observed. These redshifts further confirm the presence of oxygen or hydroxyl groups on the CNT surface [36]. The (*) marked peaks in ZnO-CNT samples are assigned to C-O bond vibrations from the acetate precursor used during synthesis [21]. Infrared spectroscopy studies of hydrogen adsorption on ZnO suggest that OH and H are adsorbed simultaneously [37]. In fact, the dissociation of hydrogen followed by its adsorption manifests as a change in the corresponding vibrational frequency, including stretching vibrations of Zn-H and O-H, which are very different from the free hydroxyl group vibrational frequency [38]. However, hydrogen adsorption is more likely on prismatic surfaces, implying that facetted ZnO nanoparticles would be more susceptible to hydrogen adsorption [39]. However, for successful hydrogen adsorption, firstly, a more acidic environment is required when working in aqueous media, or a high pressure when working in gaseous media. In addition, the nanoparticles presented in this study are spherical and not facetted. In our case, the NaOH-rich conditions provide a basic environment that is advantageous to the adsorption of hydroxyl groups, further promoted by the presence of V_o_.

Figure 5c shows the Raman spectra of ZnO-PEDOT:PSS samples in the range (900–1700 cm^−1^), where the contributions from PSS and PEDOT vibrational modes are the most significant. Two typical PSS vibrational modes at 988 cm^−1^ and 1097 cm^−1^ are observed [40]. The vibrational modes of PEDOT observed at 1263 cm^−1^, 1369 cm^−1^, 1436 cm^−1^ and 1517 cm^−1^ correspond to C_α_-C_α_, C_β_-C_β_, symmetrical C_α_=C_β_ and asymmetrical C_α_=C_β_ stretching vibrational modes, respectively. In the ZnO-D-PEDOT:PSS samples, the symmetrical vibrational mode at 1436 cm^−1^ is redshifted compared to the pristine PEDOT:PSS (~1440 cm^−1^) [41]. However, this mode is slightly more redshifted in the ZnO-A-PEDOT:PSS, suggesting a slightly higher benzoid (coil) to quinoid (linear) structural transition [41,42]. The PEDOT chains of linear conformation tend to increase the conductivity of the polymer due to a stronger covalent bonding with ZnO. Additionally, asymmetrical C_α_=C_β_ bonds of PEDOT have similar intensities for both samples, whereas, C_α_-C_α_ and C_β_-C_β_ bonds for sample ZnO-D-PEDOT:PSS are more intense than sample ZnO-A-PEDOT:PSS. On the other hand, the asymmetrical C_α_=C_β_ bond of PEDOT at 1517 cm^−1^ is more intense for ZnO-A-PEDOT:PSS samples. This implies that the bonds in the PEDOT chain have undergone structural modification provoking a breakdown in symmetry. This again suggests that PEDOT was degraded or removed from the macromolecule upon combining with ZnO-A.

### 3.2. Optical Properties

**Figure 6:**
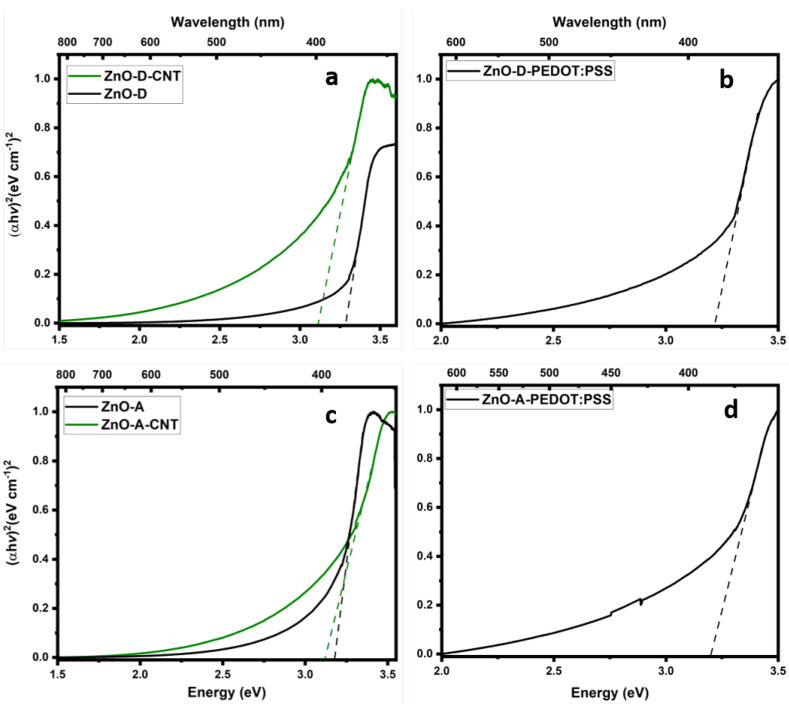
Tauc plot of (**a**) ZnO-D and ZnO-D-CNT, (**b**) ZnO-D-PEDOT:PSS, (**c**) ZnO-A and ZnO-A-CNT, and (**d**) ZnO-A-PEDOT:PSS.

The band gaps of the ZnO samples, ZnO-CNT hybrids and ZnO-PEDOT:PSS hybrids were calculated via UV-Vis absorption spectroscopy followed by Tauc plots, presented in Figure 6. The band gaps of these samples range from 3.11 to 3.3 eV, which correspond to the theoretical band gap of ZnO, implying that the absorbance in the nanocomposites is dominated by ZnO. Depending on the synthesis routes, variations in the band gaps of ZnO have been observed [43]. The absorption spectra of ZnO samples revealed a sharp shoulder at ~3.3 eV, stretching down to 2.0 eV, whereas, a broader shoulder at ~3.3 eV stretching down to ~1.5 eV was observed for the CNT hybrids [44]. 

**Figure 7:**
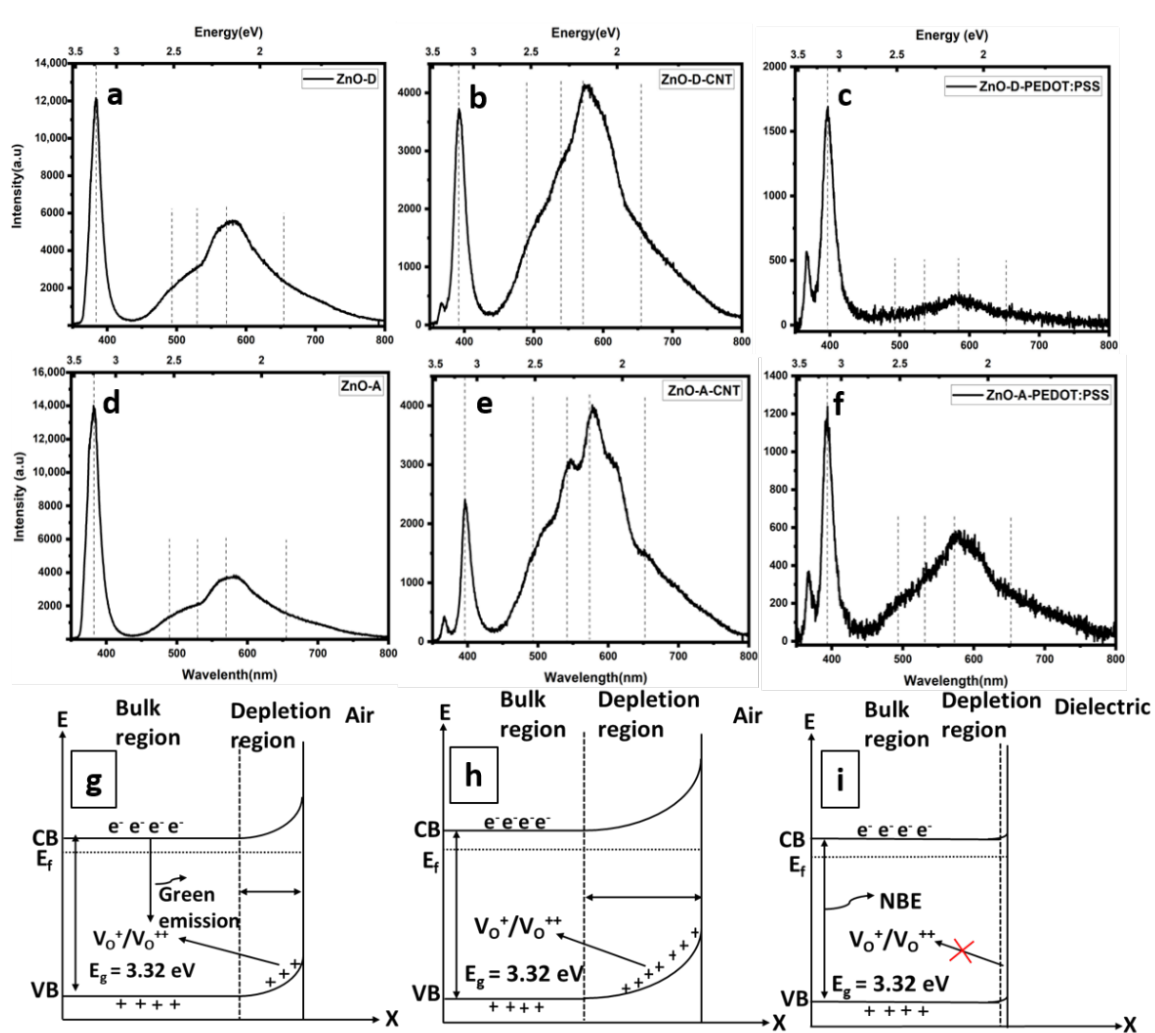
PL emission spectra of (**a**) ZnO-D, (**b**) ZnO-D-CNT, (**c**) ZnO-D-PEDOT:PSS, (**d**) ZnO-A, (**e**) ZnO-A-CNT and (**f**) ZnO-A-PEDOT:PSS. Schematics of upward band bending (**g**) chemisorbed Oxygen species, (**h**) CNT decorated with ZnO through hydroxyl groups, (**i**) ZnO-PEDOT:PSS nanohybrids.

The emission properties of the as-synthesized ZnO samples, ZnO-CNT hybrids, and ZnO-PEDOT:PSS hybrids were investigated at room temperature. A 365 nm (3.4 eV) excitation source was used to induce band-to-band transitions in these samples with band gaps between 3.11 eV and 3.3 eV. The PL spectra in Figure 7a–f present typical PL emission characteristics of ZnO nanoparticles, consisting of the NBE and DLE [45]. The similarities in emission peak localizations indicate that the emissions mainly originate from ZnO nanoparticles, for both the freestanding and hybrid nanocomposites. However, there are significant changes in the overall quantum efficiencies and intensities of certain emission peaks of the hybrid samples. This suggests that interfacial bonding between ZnO nanoparticles and CNT or PEDOT:PSS via OH groups plays an important role in excitonic separation and recombination. The probable origin of the DLE is the combination of several point defects, such as oxygen interstitials (O_i_), oxygen vacancies (V_O_), zinc vacancies (V_Zn_), zinc interstitials (Zn_i_), and their complexes [22,46] that are related to the presence of hydrates in the ZnO precursor. In addition, the NBE to DLE ratio is useful in evaluating the crystalline quality of ZnO. Moreover, an average nanoparticle size of 5 nm indicates a high surface-to-volume ratio, which in turn denotes a high amount of surface defects. These surface traps also consist of chemisorbed species, allowing additional radiative or non-radiative recombination mechanisms, which would alter the quantum efficiency of the DLE. 

The doubly ionized oxygen vacancy, i.e., V_O_^++^, or surface oxygen vacancy at 2.2 eV is dominant in all the samples due to the high surface-to-volume ratio. The 2.2 eV transition is associated with the capture of a hole by V_O_^+^ from surface charges to form V_O_^++^ [47]. The single ionized oxygen vacancy V_O_^+^ or volume oxygen vacancy emits at ~2.5 eV. Both types of vacancies produce green luminescence in ZnO. Additionally, the intensity of the green emission can be strongly influenced by free carriers on the surface, especially for nanoparticles with very small sizes [47,48]. Since PL measurements were performed in air, it is likely that hydroxyl groups or oxygen molecules are adsorbed on the surface of the nanoparticles. The chemisorbed oxygen species provoke an upward band bending in the as-synthesized ZnO nanoparticles (Figure 7g), which allows V_O_^+^ to convert into V_O_^++^ through the tunneling of surface-trapped holes to deep levels. Therefore, the observed dominant green emission in ZnO-D and ZnO-A samples is mainly surface related. In a previous study, the chemisorption of hydroxyl groups/oxygen species was suppressed by covering the nanoparticles with CNT [17]. In that study, non-functionalized CNT were used, and a successful passivation of surface states was obtained. In the present case, the CNT were functionalized with OH functional groups, as discussed previously. Therefore, in the present case, the upward band bending is enhanced (Figure 7h). The increased upward band bending leads to further increase in the depletion region size, whereupon the probability of electron capture at the defect sites increases. This mechanism also reduces the probability of band-to-band transitions and the NBE is diminished. 

For ZnO-PEDOT:PSS samples, a complete coverage of ZnO with PEDOT:PSS is visible in the TEM images. In general, there is a reduction in the overall emission compared to the as-synthesized and ZnO-CNT samples, due to the low amount of ZnO nanoparticles. However, the NBE-to-DLE ratio is higher in these samples. The increase in NBE can be attributed to the reduced surface hydroxyl groups, relative to the as-synthesized and ZnO-CNT samples, leading to lower upward band bending (Figure 7i). More particularly, the NBE-to-DLE ratio is higher for the ZnO-D-PEDOT:PSS than ZnO-A-PEDOT:PSS. An increase in DLE for the latter can be attributed to the higher amount of hydroxyl groups present on the ZnO-A sample, as assessed on the basis of the XPS studies, leading to a slightly higher upward band bending than for ZnO-D. Additionally, the NBE of both types of hybrid sample, i.e., CNT and PEDOT:PSS, has redshifted, suggesting an increased amount of Zn_i_, further corroborating the Raman spectroscopy results. Finally, the red emission at ~1.75 eV is the least significant component in the PL spectra, associated mainly with V_Zn_-related defects [49].

## 4. Conclusions

In this study, we successfully synthesized ZnO nanoparticles and their hybrids containing CNT and PEDOT:PSS. The effect of hydroxyl groups on the optical properties of ZnO nanoparticles and their hybrids was investigated. The ZnO nanoparticles display optical properties that are both bandgap and defect related. In addition, the choice of precursor immensely influences the overall properties of the nanoparticles and their hybrids. During the synthesis of the hybrid nanocomposites, hydroxyl groups adhere to the surface of the ZnO nanoparticles, and in turn, intensify the defect related emission. These hydroxyl groups are necessary for the successful decoration of the CNT and the incorporation of ZnO in the PEDOT:PSS matrix. Additionally, the linear transformation of PEDOT from the coil structure implies a more conductive polymer, which would enhance the I-V characteristics of the nanocomposite. However, ZnO-A tends to degrade the PEDOT:PSS macromolecule by removing or degrading the conducting PEDOT, which could prove detrimental to the electrical properties of the nanocomposite. Future research consists of incorporating these nanoparticles and their hybrids in LED applications. This present work aids in understanding the modification of the physical and chemical properties of the ZnO nanoparticles when hybridized to PEDOT:PSS and CNT. On the basis of this work, we conclude that ZnO-D and its hybrid nanocomposites, synthesized with hydrate precursors show higher stability and are likely to offer better electrical conductivity when used in LED.

## Figures and Tables

**Table 1:** List of ZnO-CNT hybrids and ZnO-PEDOT:PSS hybrids synthesized in this work.

Sample Name	CNT (wt%)	PEDOT:PSS (wt%)
ZnO-D-CNT	~1	-
ZnO-A-CNT	~1	-
ZnO-D-PEDOT:PSS	-	~95
ZnO-A-PEDOT:PSS	-	~95

## Data Availability

Not applicable.
